# Preparation and characterization of a highly stable phenoxazinone synthase nanogel

**DOI:** 10.1186/s13065-016-0178-8

**Published:** 2016-05-28

**Authors:** Honghua Jia, Zhen Gao, Yingying Ma, Chao Zhong, Chunming Wang, Hua Zhou, Ping Wei

**Affiliations:** College of Biotechnology and Pharmaceutical Engineering, Nanjing Tech University, Nanjing, 211800 China

**Keywords:** Phenoxazinone synthase, Laccase, Nanogel, Stability, Solvent resistance

## Abstract

**Background:**

Phenoxazinone synthase (PHS) is a laccase-like multicopper oxidase originating from *Streptomyces* with great industrial application potential. In this paper, we prepared the PHS nanogel retaining 82 % of its initial activity by aqueous in situ polymerization at pH 9.3.

**Results:**

The average diameter of the PHS nanogel was 50.8 nm based on dynamic light scattering (DLS) analysis. Fluorescence analysis indicated the impressive preservation of the enzyme molecular structure upon modification. The PHS nanogel exhibited the most activity at pH 4.0–4.5 and 50 °C while the corresponding values were pH 4.5 and 40 °C for the native PHS. The *K*_m_ and *V*_max_ of the PHS nanogel were found to be 0.052 mM and 0.018 mM/min, whereas those of the native PHS were 0.077 mM and 0.021 mM/min, respectively. In addition, the PHS nanogel possessed higher thermal and storage stability and solvent tolerance compared with the native one. The half-life of the PHS nanogel was 1.71 h and multiplied around ninefold compared to 0.19 h for the native one.

**Conclusion:**

In summary, the PHS nanogel could be a promising biocatalyst in industry.

## Background

Phenoxazinone synthase (PHS, EC 1.10.3.4) is a bacterial laccase-like multicopper oxidase firstly described by Katz and Weissbach [1]. As a key enzyme for actinomycin D biosynthesis in *Streptomyces*, the properties of PHS were preliminarily characterized originally by Golub and Nishimura [2]. They found it can catalyze oxidation of catechols, ferrocyanide, and ethylenic thiols, in addition to *o*-aminophenols, which was similar to laccase. In general, PHS exists in a hexameric form which exhibits the most activity [3]. In consideration of its catalytic properties, PHS is a promising enzyme for use in antibiotics production, dye synthesis, bio-bleaching, and bio-detoxication [4–7].

Owing to lower stability, enzymes usually fail to meet the need of industrial processes. For a long time, chemical modification of key groups has enabled enzyme improvement in terms of stability and other features [8–10]. Unlike the other methods, chemical modification can unlimitedly alter side chain of amino acid structures without the need of sequence or structure information [11]. Chemical modification might strengthen the intrinsic rigidity of the molecule to enhance pH and temperature stability and organic solvent tolerance [8, 12].

In recent years, enzyme modification on a nanoscale is drawing more and more attention for its ability to confer higher activity and stability [13, 14]. The soluble single-enzyme nanoparticles (SENs) of α-chymotrypsin and trypsin have been prepared by surrounding enzyme molecule with a nanometer thick porous composite organic/inorganic network, and exhibited impressive stability with minimal substrate mass-transfer limitation [15]. After that, the SENs has been embedded into nanoporous silica and showed higher operational stability [16]. Besides, several similar enzyme nanogels involving horseradish peroxidase, lipase, carbonic anhydrase and laccase have been synthesized by using an innovative aqueous in situ polymerization with excellent thermal stability and tolerance resistance [17–21]. The possible mechanism for improving stability has also been proposed by molecular simulation [22, 23].

In the present study, for the purpose of improving the properties, we prepared the PHS nanogel via in situ polymerization. The resultant PHS nanogel was analyzed by SEC, and fluorescence analysis. Subsequently, kinetic parameters, thermal and storage stability, and solvent tolerance were also characterized in detail.

## Results and discussion

### Effect of pH on the modification

**Fig. 1:**
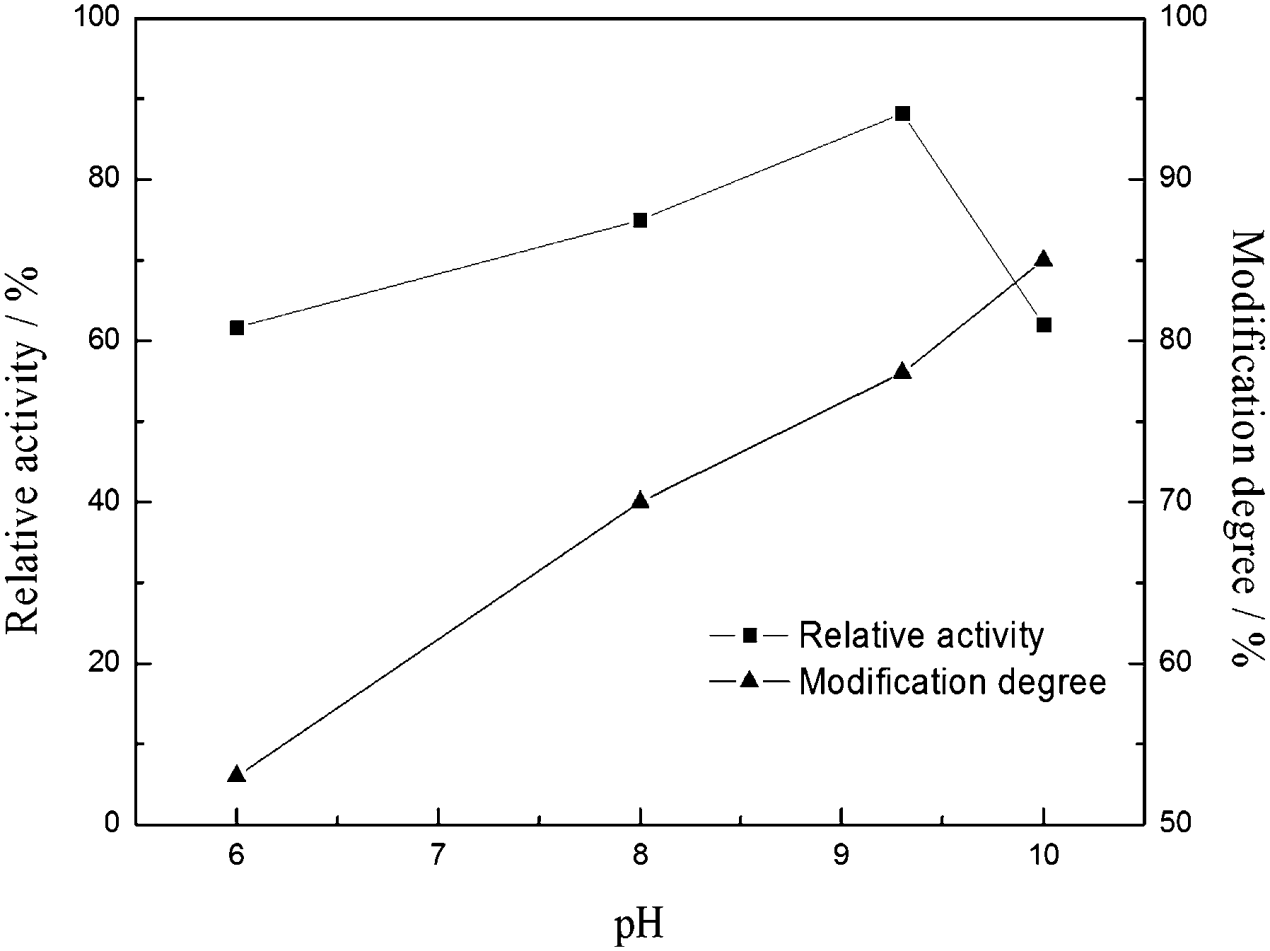
Effect of pH on the modification of the PHS

The modification yield of PHS by NAS would be altered with respect to pH. The modification yield and activity of PHS increase gradually with the rise of pH below 9.3 as is presented in Fig. 1. Upon above pH 9.3, the modification yield mounts continually, whereas the activity decreases. It is apparent that around 90 % of its initial activity can be kept with 78 % of modification yield at pH 9.3. The enhancement of modification yield could be visibly credited to the increase in capability of nucleophilic attack of amino group for readily deprotonating at higher pH. On the other hand, the decrease in activity resulted from slight change in tertiary structure of enzyme with the generation of new ionic bridges or interactions for change in charged groups with the modification on amino groups [12].

### Effect of concentration of acrylamide on PHS nanogel preparation

**Fig. 2:**
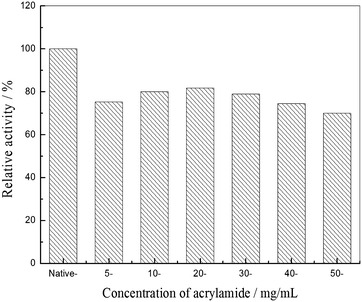
Effect of concentration of acrylamide on the PHS nanogel preparation

The influence of acrylamide on PHS nanogel preparation was probed at concentration of acrylamide in the range 5–50 mg/mL, and the results are shown in Fig. 2. It can be found that approximately 82 % of its initial activity was remained at 20 mg/L of acrylamide. When the concentration of acrylamide exceeds 20 mg/ml, the activity decreases with rising concentration of acrylamide. The decrease in activity was due to growing diffusion resistance because of forming dense gel grid at higher concentration of acrylamide [24, 25]. In effect, diffusional limitation had been observed in the entrapment of chymotrypsin in highly crosslinked polyacrylamide gel [26]. Another reason is multipoint covalent attachment between enzyme and polyacrylamide gel network gave rise to a slight change in structure.

### DLS and fluorescence analysis

**Fig. 3:**
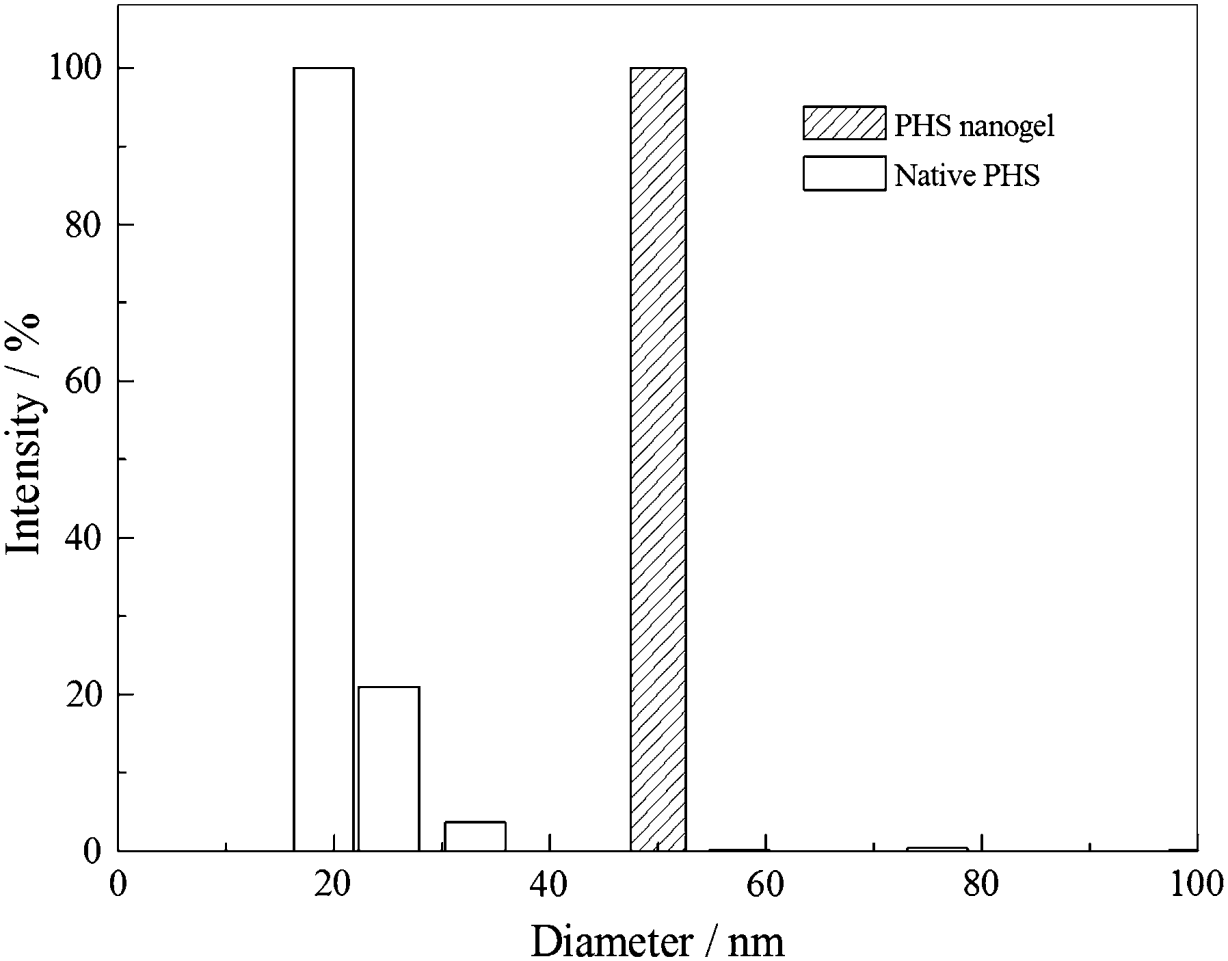
DLS analyses of the native PHS and PHS nanogel

**Fig. 4:**
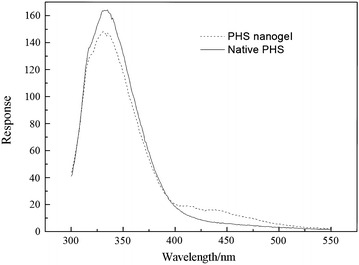
Fluorescence spectra of the native PHS and PHS nanogel

As is displayed in Fig. 3, DLS analyses indicated that the diameter of the native PHS was ranging from 19.03–33.1 nm with an average 20.8 nm. Compared to the native one, the diameter of the PHS nanogel appears a fairly uniform distribution with an average 50.8 nm. Fluorescence emission spectra of the native PHS and PHS nanogel are shown in Fig. 4. The maximal fluorescence emission wavelength of the native PHS and PHS nanogel at around 330 nm indicates that there was no significant change of the enzyme molecular structure upon modification. The observations were in accord with other results in previous studies [22].

### Optimum pH and temperature

**Fig. 5:**
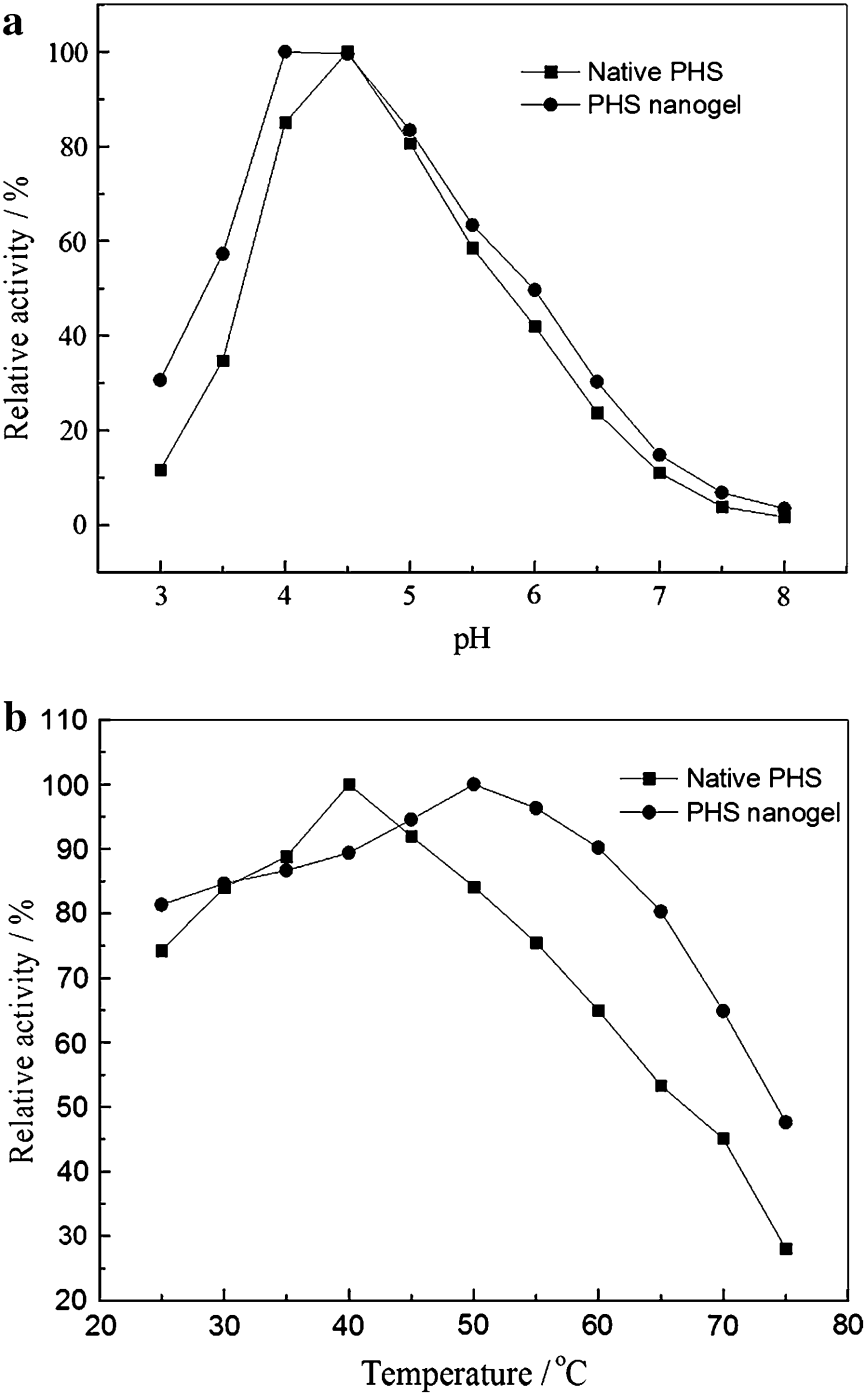
Effect of pH and temperature on the native PHS and PHS nanogel. **a** pH; **b** Temperature

The effect of different pH on the activity of the native PHS and PHS nanogel was investigated at pH ranging from 3.0 to 8.0 (Fig. 5a). The results signified that the PHS nanogel showed maximum activity at pH 4.0–4.5 as compared to the native one that showed maximum activity at pH 4.5. There was no significant change in the pH optimum of the enzymes, indicating that there was no distinct influence caused by slight alteration in conformation on the enzymes during nanogel preparation.

The temperature profiles of the native PHS and PHS nanogel were also examined over a temperature range from 25 to 75 °C. As can be seen from Fig. 5b, the native PHS reached its maximum activity at 40 °C, whereas it shifted to 50 °C for the nanogel. The shift in optimum temperature was attributed to the change on conformational flexibility as a result of formation of covalent bonds between the enzyme and the polyacrylamide gel [27].

### Kinetic parameters

**Fig. 6:**
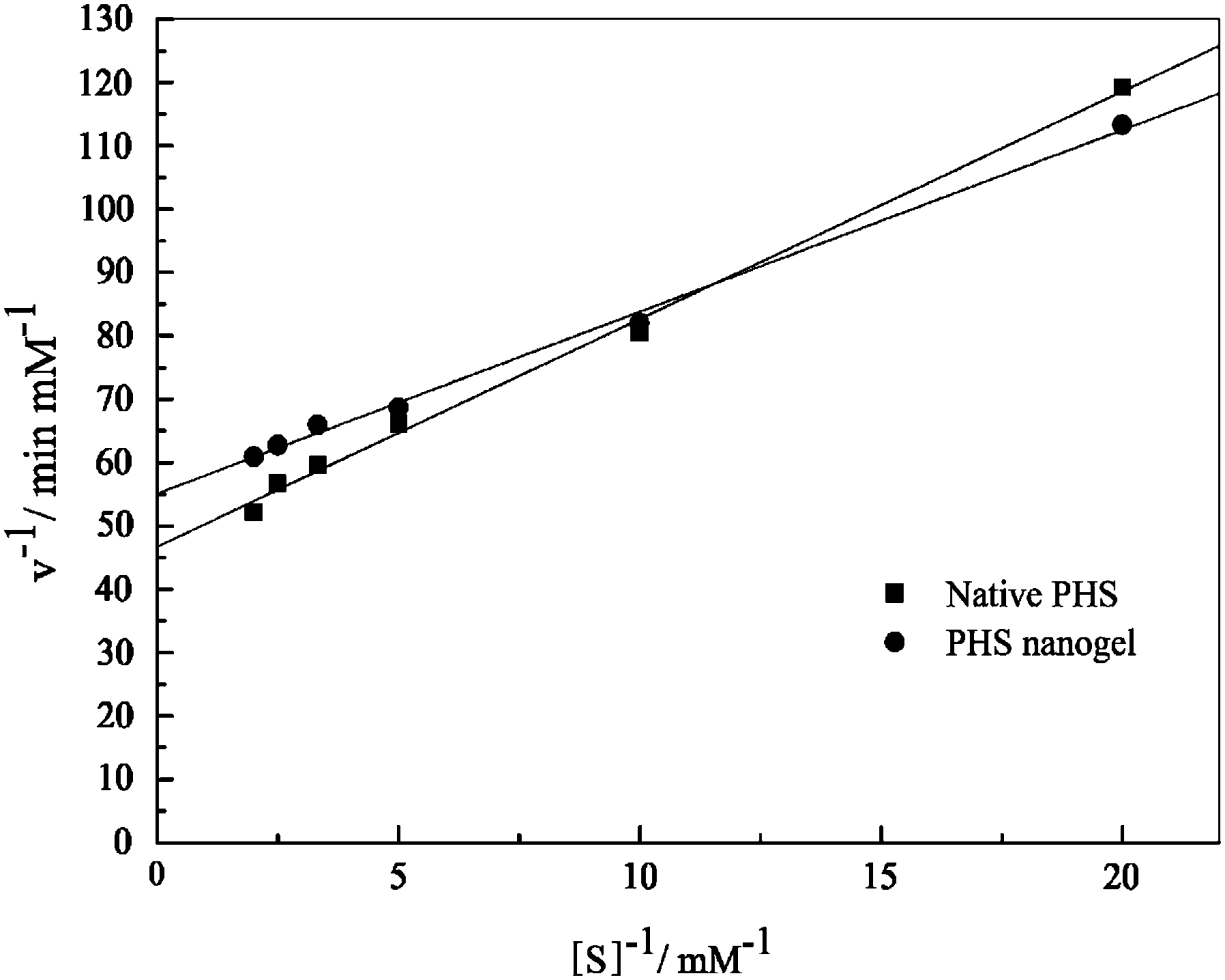
Lineweaver-Burk *plot* of the native PHS and PHS nanogel

The kinetic parameters of the native PHS and PHS nanogel are summarized in Table 1, which were calculated from the Lineweaver–Burk plot (Fig. 6). The *K*_m_ of the native PHS was 0.077 mM, while it was 0.052 mM for the PHS nanogel, approximately 20 % lower than that of the native one, which means the PHS nanogel has higher affinity towards the substrate. Similar phenomena were also observed in other studies on CLEA and nanogel of laccase [28, 29]. The decrease in *K*_m_ might be caused by the slight conformational change of the active site necessary for substrate binding after modification of PHS. In addition, the partition of substrate on the enzyme environment is also responsible for that. As to *V*_max_, it was decreased from 0.021 mM/min of the native PHS to 0.018 mM/min of the PHS nanogel. It was supposed that both the slight conformational change and the increasing mass transfer resistance could be responsible for the decrease in *V*_max_ [30].

**Table 1:** Kinetic parameters of the native PHS and PHS nanogel

	Equation	*K*_m_/mM	*V*_max_/mM/min
Native PHS	*v* ^−1^ = 3.59[*S*]^−1^ + 46.73 (*R* ^2^ = 0.9981)	0.077	0.021
PHS nanogel	*v* ^−1^ = 2.87[*S*]^−1^ + 55.11 (*R* ^2^ = 0.9984)	0.052	0.018

### Thermal stability

**Fig. 7:**
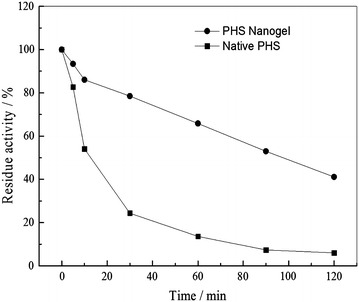
Thermal stability of the native PHS and PHS nanogel

Thermal stability of enzyme is one of the most important criteria for its application. Here, thermal stability of the native PHS and PHS nanogel was tested by incubating at 60 °C and enzyme activity was measured at different time intervals as described above. It can be found that the native PHS lost about 90 % of its activity whereas the PHS nanogel lost about 50 % of its activity for 2 h preincubation, as is shown in Fig. 7. According to the curve given in Fig. 7, the calculated half-life of the PHS nanogel was 1.71 h and had multiplied around ninefold compared to 0.19 h for the native one. It was demonstrated that the thermal stability of enzymes would be drastically increased if attached to a relatively rigid support [31]. There are many factors affecting the stability of enzyme. Firstly, many previous instances showed that chemical modification of key groups of enzyme was very important to the stability of enzyme [32]. For instance, in vivo methylation of lysyl residues of enzyme has been revealed to be crucial for thermal stability of enzyme [33, 34]. Secondly, research had showed that protein oligomerization could play a major role in thermal stability for the lower mobility of the groups in the subunit–subunit multi-interactions [35]. In the PHS nanogel, the multi-interactions between subunits would be higher order and the association as well as dissociation of subunits would be prevented due to the multipoint covalent attachment, which is potentially important for enhancing the stability [36–38]. Finally, the multipoint covalent attachment between PHS and polyacrylamide would keep a strong structure rigidification to prevent enzyme conformational changes when the conditions are altered [39, 40].

### Solvent resistance

**Fig. 8:**
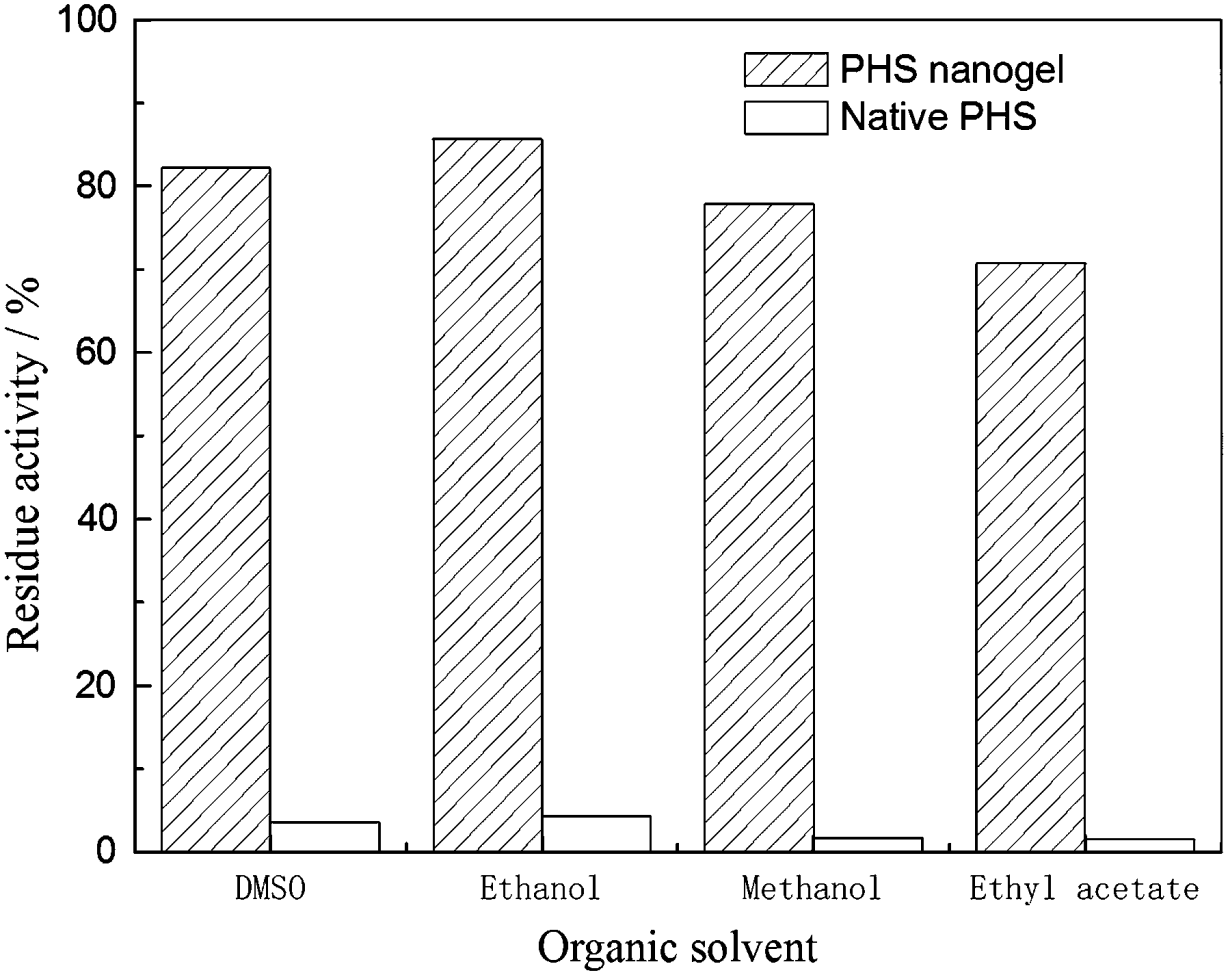
Organic solvents tolerance of the native PHS and PHS nanogel

The PHS nanogel exhibited better stability than the native one in organic solvents. As is presented in Fig. 8 the native PHS would clearly maintain less than 5 % of its activity in all tested solvents, while the activity could remain at least 70 % for the PHS nanogel. The possible reasons accounting for the increase in solvent tolerance of the PHS nanogel were listed as follows: (1) The increased intrinsic rigidity of enzyme with covalent attachment on polyacrylamide gel [41]; (2) The polyacrylamide gel can maintain a hydrophilic shell for PHS molecule's surface which could restrain the loss of essential water of enzyme molecules and decrease the organic solvent concentration in the microenvironment [42, 43].

### Storage stability

**Fig. 9:**
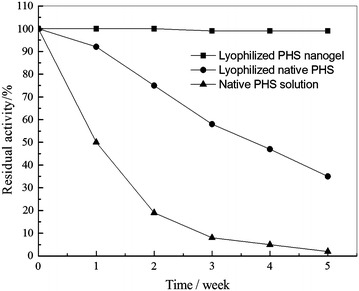
Storage stability of the PHS solution, lyophilized native PHS and lyophilized PHS nanogel

Generally, enzyme activity will decrease gradually by time during storage. Therefore, storage stability is usually considered as one of the significant indexes to evaluate enzyme properties. As is shown in Fig. 9, the lyophilized PHS nanogel was apparently more stable than the PHS solution and lyophilized PHS stored at 4 °C. The PHS solution and lyophilized PHS lost its 98 and 65 % activity when stored at 4 °C for 5 weeks while the PHS nanogel retained nearly 100 % of its initial activity. The higher storage stability of the PHS nanogel could be explained as the prevention of structural denaturation as a result of the encapsulation of PHS by polyacrylamide [44].

## Experimental section

### Materials

PHS was prepared according to the previous publication [45]. *N*-Acryloxysuccinimide (NAS), 2, 2′-azino-bis (3-ethyl benzothiazoline-6-sulfonic acid) diammonium salt (ABTS) and 2, 4, 6-trinitrobenzenesulfonic acid solution (TNBS) were purchased from Sigma-Aldrich (Shanghai, China). Tetramethylethylenediamine (TEMED), acrylamide, ammonium persulfate and trehalose were supplied by Sinophar Chemical Reagent Co., Ltd (Shanghai, China). All other chemicals used were of analytical grade.

### The preparation of PHS nanogel

**Fig. 10:**
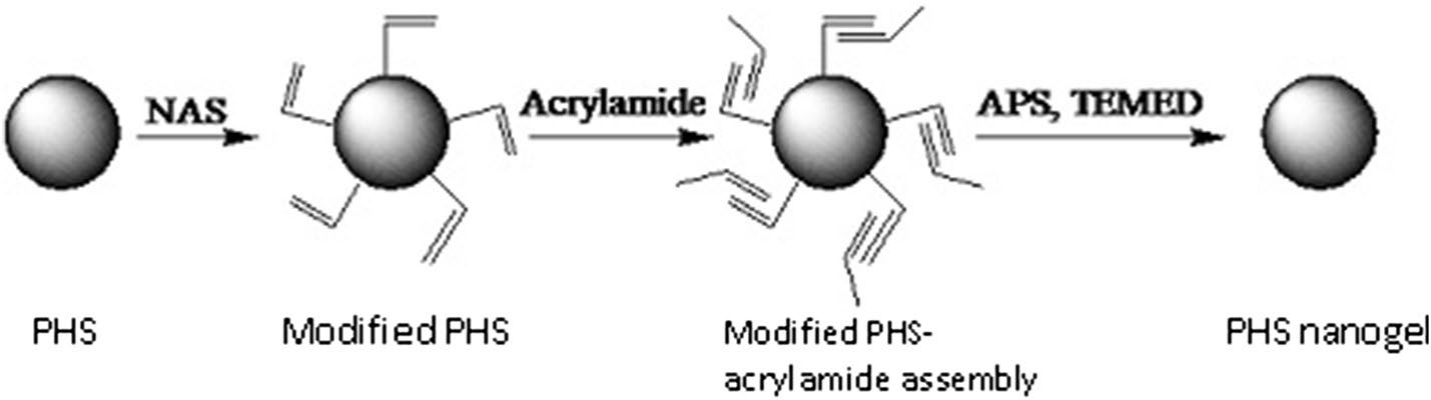
Scheme of preparation of the PHS nanogel

The PHS nanogel was prepared by aqueous in situ polymerization as previously described [21]. Five milliliter of PHS solution was dialyzed against borate buffer (50 mM, pH 9.3). 10 mg of NAS dissolved in 600 μL of DMSO, was dropwise added to the PHS solution. After 4 h reaction at 30 °C with agitation, the mixture was dialyzed against phosphate buffer (50 mM, pH7.0) at 4 °C for 36 h. Later on, 20 mg of acrylamide was added after N_2_ purging for 30 min, and 15 mg of ammonium persulfate and 15 μL of TEMED were added to initiate polymerization under N_2_ purging at 30 °C for 12 h (Fig. 10). The product solution was then subjected to dialysis against phosphate buffer (50 mM, pH 7.0) for 24 h and deionized water for another 2 h at 4 °C to remove unreacted reagents, and resulting to PHS nanogel by lyophilization with the addition of trehalose to 2 %.

### Determination of modified amino group

The sulfonate group of TNBS can react specifically with the free amino groups of proteins and the resulting derivatives can be determined spectrophotometrically. TNBS method is usually used for the determination of free amino groups in proteins [46, 47]. In this paper, the modified amino group in the PHS preparation was determined by using the TNBS method, and the modification yield was defined as the ratio of modified amino groups in protein.

### DLS analysis

The DLS analysis of the native PHS and PHS nanogel was conducted at 25 °C on a Brookhaven BI-200SM laser light scattering system with a 90° scattering angle.

### Fluorescence analysis

The fluorescence analyses of the native PHS and PHS nanogel excited at 285 nm were recorded from 300 to 550 nm with a Shimadzu RF-5301 PC spectrofluorometer.

### Determination of PHS activity

The native PHS and PHS nanogel activity was determined spectrophotometrically by monitoring the increase in absorbance at 420 nm of a reaction mixture containing 0.5 mM ABTS in 0.1 M sodium acetate buffer (pH 4.5) and a suitable amount of enzyme at 25 °C [45]. One unit of PHS activity was defined as the amount of enzyme oxidizing 1 μmol of ABTS per minute (ε_420_ = 36 mM^−1^ cm^−1^).

### Optimum pH and temperature

To investigate the optimum pH and temperature of the native PHS and PHS nanogel, the activity of the native PHS and PHS nanogel was measured using ABTS as substrate at pH (3.0–8.0) and temperature (25–75 °C), respectively.

### Kinetic parameters

The kinetic parameters, *K*_m_ and *V*_max_, of the native PHS and PHS nanogel were calculated by the Lineweaver–Burk plot. Reactions were conducted based on the determination of activity method using 0.05–0.5 mM ABTS.

### Thermal stability

The native PHS and PHS nanogel stabilizing against thermal denaturation were tested in acetate buffer (100 mM, pH 4.5) at 60 °C and the activity was determined after sampling periodically as described above. The residual activity was expressed as the percentage with respect to initial activity.

### Solvent resistance

The investigations into solvent tolerance of the native PHS and PHS nanogel were carried out by incubating in different organic solvents at 30 °C for 1 h. Then the activities were assayed as described above.

## Conclusions

In this paper, a designed nanogel prepared by aqueous in situ polymerization at pH 9.3, which could retain 82 % of PHS activity was introduced. The average diameter of the PHS nanogel was 50.8 nm based on dynamic light scattering analysis. Fluorescence analysis indicated the impressive preservation of the enzyme molecular structure upon modification. The PHS nanogel exhibited the most activity at pH 4.0–4.5 and 50 °C while the corresponding values were pH 4.5 and 40 °C for the native PHS. The *K*_m_ and *V*_max_ of the PHS nanogel were found to be 0.052 mM and 0.018 mM/min, whereas those of the native PHS were 0.077 mM and 0.021 mM/min, respectively. In addition, the PHS nanogel had possessed higher thermal and storage stability and solvent tolerance compared with the native one. The half-life of the PHS nanogel was 1.71 h and had multiplied around ninefold compared to 0.19 h for the native one.

It is the first investigation into the nanogel preparation and characterization of PHS (phenoxazinone synthase) originated from *Streptomyces* in this paper. Based on the enzymatic properties were characterized in detail, results showed that the resultant PHS nanogel have indicated higher thermal and storage stability and solvent resistance. As a result, the PHS nanogel could be a promising biocatalyst in industry.

## Abbreviations

ABTS: 2, 2′-azino-bis (3-ethyl benzothiazoline-6-sulfonic acid) diammonium salt
DLS: dynamic light scattering
NAS: *N*-acryloxysuccinimide
PHS: phenoxazinone synthase
TEMED: tetramethylethylenediamine
TNBS: 2, 4, 6-trinitrobenzenesulfonic acid solution
